# A Fault Detection Approach Based on One-Sided Domain Adaptation and Generative Adversarial Networks for Railway Door Systems

**DOI:** 10.3390/s23249688

**Published:** 2023-12-07

**Authors:** Minoru Shimizu, Yifan Zhao, Nicolas P. Avdelidis

**Affiliations:** 1 Integrated Vehicle Health Management Centre, Cranfield University, Cranfield MK43 0AL, UK; np.avdel@cranfield.ac.uk; 2 Centre for Life-Cycle Engineering and Management, Cranfield University, Cranfield MK43 0AL, UK; yifan.zhao@cranfield.ac.uk

**Keywords:** data-driven approach, deep learning, domain adaptation, door systems, fault detection, generative adversarial network, machine learning, railway

## Abstract

Fault detection using the domain adaptation technique is one of the more promising methods of solving the domain shift problem, and has therefore been intensively investigated in recent years. However, the domain adaptation method still has elements of impracticality: firstly, domain-specific decision boundaries are not taken into consideration, which often results in poor performance near the class boundary; and secondly, information on the source domain needs to be exploited with priority over information on the target domain, as the source domain can provide a rich dataset. Thus, the real-world implementations of this approach are still scarce. In order to address these issues, a novel fault detection approach based on one-sided domain adaptation for real-world railway door systems is proposed. An anomaly detector created using label-rich source domain data is used to generate distinctive source latent features, and the target domain features are then aligned toward the source latent features in a one-sided way. The performance and sensitivity analyses show that the proposed method is more accurate than alternative methods, with an F1 score of 97.9%, and is the most robust against variation in the input features. The proposed method also bridges the gap between theoretical domain adaptation research and tangible industrial applications. Furthermore, the proposed approach can be applied to conventional railway components and various electro-mechanical actuators. This is because the motor current signals used in this study are primarily obtained from the controller or motor drive, which eliminates the need for extra sensors.

## 1. Introduction

Fault detection plays a vital role in maintenance tasks within the railway sector, and has been defined as “the detection of a fault within a prescribed time by a safety mechanism” [1]. Although railway machinery represents a complex industrial system with a huge variety of components, a train door is a vital subsystem that can lead to service interruptions or failures, resulting in higher operational and maintenance expenses. One report has indicated that the door system accounts for 30–60% of all malfunctions in railway vehicles [2]. To avoid such failures, predictive maintenance using data-driven methods has recently gained interest from researchers, owing to the vast quantities of monitoring data accessible.

Data-driven approaches for fault detection include traditional machine learning (ML) and deep learning (DL) approaches. Traditional ML approaches necessitate multiple steps such as data preprocessing and feature extraction prior to model development; however, manual feature extraction requires specialised domain expertise, which complicates the use of traditional machine learning methods. In contrast, DL methods allow for the development of fault detection models without the need for manually crafted features by employing a deep network architecture. This represents a notable advantage over traditional ML techniques.

Fault detection methods based on deep learning can be divided into supervised and unsupervised learning approaches. Supervised DL methods require datasets with labels to enable the training of the model. A significant proportion of prior research in the area of fault detection has focused on supervised DL methods, including deep neural networks (DNNs) [3], two-dimensional convolutional neural networks (2D CNNs) [4], one-dimensional convolutional neural networks (1D CNNs) [5], gated recurrent units (GRUs) [6], and long short-term memory (LSTM) [7]. However, the need for ample labelled datasets represents a major limitation of supervised methods: faulty data are often scarce, due to the use of conservative maintenance schedules to prevent major incidents. In addition, the imbalance between faulty and healthy data is also problematic when building a classifier. Unlike supervised approaches, unsupervised DL methods do not need datasets with labels, and the aim is to extract the relevant characteristics of the input data. Previous research based on unsupervised learning approaches has included stacked autoencoders [8], denoising autoencoders [9], sparse autoencoders [10], variational autoencoders [11], and deep belief networks [12].

Despite the successful outcomes of previous research, one significant drawback of a data-driven fault detection approach is that the fault detection performance may be considerably degraded when the model is applied to actual acquired data rather than training data. The assumption underlying traditional ML and DL approaches is that the distribution of the test data is identical to that of the training data; however, these distributions may differ due to the different operating conditions, components, and detailed specifications of the machinery. This is known as the domain shift problem [13], which refers to the discrepancy in the feature distribution between two domains. In this case, the training and test data are assumed to be the source and target domains, respectively. Due to the discrepancy between the source and target domains, the accuracy of fault detection in actual industrial data may be worse than anticipated. If the plan is to acquire many types of data beforehand, under different operating conditions and for different components, then the training of the model may be expensive and demanding, meaning that a huge dataset would be required. This strategy is therefore impractical, given that the availability of datasets that include sufficient faulty samples is always limited in industry.

The domain adaptation (DA) technique offers a promising solution for addressing the domain shift problem. DA is an area of machine learning where models are designed to execute tasks in a target domain, using knowledge gained from a similar but distinct source domain. The aim is to address the challenge posed by the distribution shift between the source and target domains. Research into DA has evolved over the years in several areas of study, including computer vision, healthcare, speech recognition, and fault detection. When applied in the context of fault detection, the aim of DA is to align the source and target domain distributions. The aligned source and target features are then used to build a fault detection model. As a result, faults can be detected with almost the same accuracy for both the source and target domains.

Fault detection based on DA can be categorised at the methodological level into network-based, instance-based, mapping-based, and adversarial-based approaches [14]. Network-based DA involves the direct transfer of certain network parameters that have been pre-trained in the source domain to another model in the target domain, as partial network parameters. The fine-tuning of the network parameters is then conducted using a limited set of labelled data from the target domain [15,16]. However, sufficient labelled target domain datasets are necessary for this method, which are often unavailable in the context of fault detection. Instance-based DA involves adjusting the weights of instances in the source domain to assist the classifier in label prediction or using instance statistics to bring the target domain into alignment. These methods include DNNs with a batch normalisation layer (BN) [17] and adaptive batch normalisation [18,19]. However, in order to train the BN layer and set appropriate parameters, a certain amount of normal and faulty target samples is required beforehand.

The aim of mapping-based DA is to project the original features from both the source and target domains into a new feature space, where the two domain features are aligned using a feature extractor. There are many examples of fault detection using mapping-based DA, including Kullback–Leibler divergence [20], correlation alignment (CORAL) [21], maximum mean discrepancy (MMD) [22,23], multi-kernel MMD [24,25], and joint distribution adaptation [26]. In contrast, adversarial-based DA uses an adversarial method in which a domain discriminator is used to minimise the discrepancy in the feature distribution between the source and target domains created by a feature extractor. The adversarial network architecture is called a generative adversarial network (GAN). This consists of a pair of networks that are combined to form a generative system, and was initially proposed by Ian Goodfellow in 2014 [27]. When carrying out domain adaptation with a GAN, the generator is typically employed to map the raw features from both the source and target domains to a new latent feature space, where the alignment of the feature distributions can be achieved. A fault detection model can be built using the aligned features in the latent feature space for both the source and target domains. Examples of adversarial-based DA include the domain adversarial neural network (DANN) [28,29], the domain adversarial transfer network [30], and the Wasserstein distance-based deep transfer network [31].

Adversarial-based DA can be integrated with mapping-based DA by employing both the adversarial discriminator and an appropriate objective function to minimise the discrepancy between the two domains. Research using both adversarial and mapping-based DA is being widely conducted in relation to fault detection, due to its strong ability to align two domains where only unlabelled target samples are available [31,32]. However, these methods still have some impractical aspects, which can be research gaps, as follows:

1. In a GAN, the feature generator does not take domain-specific decision boundaries into consideration, as the model simply tries to fool the discriminator [33]. This results in poor performance in terms of detecting faulty samples near the class boundary.
2. In general, the DA method causes both the source and target domain to be the same in the latent space, meaning that they are treated equally. However, the source domain information needs to be considered with a higher priority than the target domain, as the source domain tends to be a rich dataset that includes more faulty samples than the target domain under actual industrial conditions. The reason for this is that the fault detection model is initially built with a focus on the specific machinery, followed by thorough validation in order to make the model applicable to the actual industrial setting. The model is then applied to another domain.
3. Ensuring that DA techniques are robust and reliable for real-world applications is challenging if the method is completely unsupervised. This is because the degree of similarity between the two domains that is required in order to be able to apply the DA method successfully is unclear. However, reliability is crucial for fault detection to avoid catastrophic incidents; hence, real-world implementations of the DA technique are still scarce.

These are major challenges, and few studies can be found that have attempted to overcome these hurdles. In order to tackle these issues, a novel fault detection approach for railway door systems is proposed, based on one-sided DA using GANs. In this study, the source and target domains consist of data from a linear actuator test rig and a real-world railway door, respectively. First, an anomaly detector and a feature generator are trained using label-rich source domain data to generate distinctive source latent features. Next, the target domain data are aligned with the source latent features in a one-sided way. The proposed method enables the faulty target samples to be aligned with the source samples and to be detected accurately by the same anomaly detector, which is built based on rich source data. To the best of our knowledge, this paper is the first to introduce a fault detection method utilising DA specifically for railway door systems. The main contributions of the paper can be summarised as follows:

1. A fault detection approach is proposed based on one-sided DA with GANs, which can be used for real-world railway door systems.
2. The proposed one-sided DA from the target to the source domain enables the normal and faulty samples in the target domain to be detected using the same fault detection model, which is trained on a rich source dataset.
3. Our approach ensures that the two domains can be aligned, despite the low level of similarity between different components, using only a few faulty target samples.
4. The proposed method is not only the most accurate and robust among comparative models but also bridges the gap between theoretical domain adaptation research and tangible industrial applications.
5. The proposed approach can also be applied to conventional railway components and various electro-mechanical actuators. This is because the motor current signals considered in this study are primarily obtained from the controller or motor drive, thus eliminating the need for extra sensors.

The remainder of this article is organised as follows. Section 2 introduces the dataset and the proposed methodology. Some results and a discussion are given in Section 3. Finally, Section 4 concludes this article.

## 2. Materials and Methods

### 2.1. Dataset

#### 2.1.1. Linear Actuator Experimental Dataset

**Figure 1:**
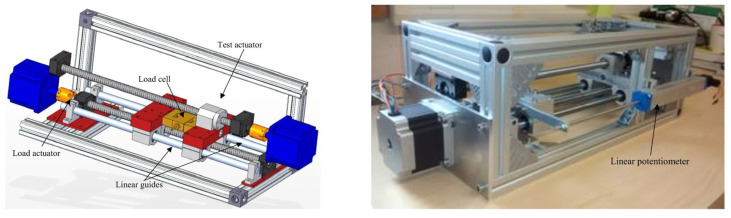
Three-dimensional model of the test rig and lateral view of the rig [36].

The primary component of the test rig was a ball screw mechanism, featuring a threaded shaft with a helical raceway that facilitated the movement of the bearing balls contained within the nut [34,35]. Different loads were produced by connecting a secondary actuator. The actuators were linked via a load cell, which supplied feedback to the controller. This setup allowed for the generation of various operating conditions by altering the load setpoint. In this case, the load setpoints used were 196.13 N, 392.3 N, and −392.3 N. Three different types of fault were introduced, with increasing severity: a lack of lubrication, spalling, and backlash. The tests were carried out using two types of motion profiles: trapezoidal (for constant speed) and sinusoidal (for smooth acceleration and deceleration). A 3D representation of the test rig, along with a side view, is presented in Figure 1. More comprehensive information about the test rig and the introduced faults is available in [34], and the raw data can be accessed and downloaded from [36].

**Figure 2:**
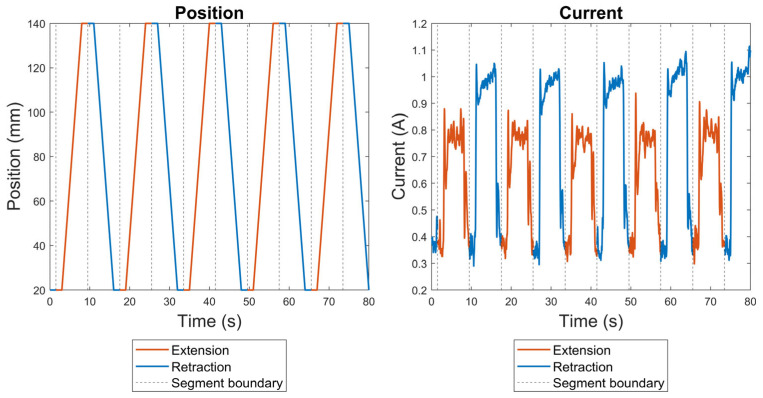
Examples of position measurements and current signals from the linear actuator test rig.

**Figure 3:**
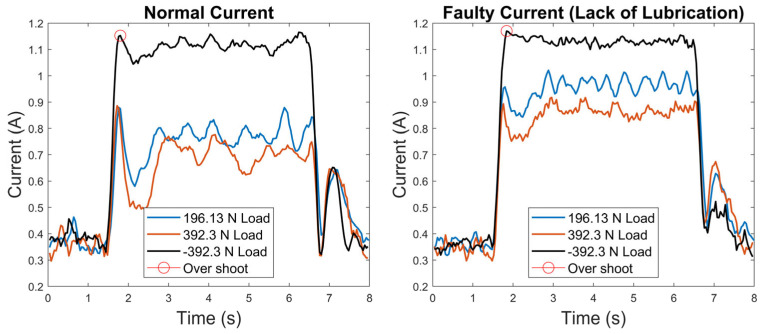
Normal and faulty current signals from the linear actuator test rig.

In our research, trapezoidal motion profiles were selected to construct the model, as railway door systems typically exhibit relatively constant speed profiles, as explained in Section 2.1.2. The measurement of the position and current signals involved both extension and retraction processes, as illustrated in Figure 2. The current signals specific to the extension operation were extracted and used to build the model, as this operation represented the closing mechanism of the railway door systems considered in this research, as explained in detail in Section 2.1.2. To reduce noise, a low-pass filter with a window of 0.15 s was applied. The current profiles that indicated a lack of lubrication were selected as the faulty current signals. Both normal and faulty current profiles are shown in Figure 3, where the various characteristics of the faulty signals can be observed; for instance, although there is some overshoot in the normal profiles, this overshoot is reduced in the faulty profiles. The dataset comprises three distinct loading conditions, which are all considered under the same class label. For example, normal profiles from these three loading conditions, as illustrated in Figure 3, are classified as the normal class, and the same categorisation applies conversely.

#### 2.1.2. Operational Datasets for Railway Door Systems

This research used extensive real-world datasets collected from railway door systems. The focus was on an electric door system consisting of a voltage power source, a DC motor, a door control unit (DCU), a transmission system, and the door leaves. The DC motor, energised by the voltage source and regulated by the DCU, delivered the required shaft angular velocity and torque, which were then conveyed to the transmission system to enable the door leaves to move in a predetermined fashion [37]. The current signal from the door was gathered via the communication port of the DCU at a frequency of 50 Hz. A low-pass filter with a 0.25 s window, equivalent to five consecutive measurement periods, was implemented to minimise the noise in the current signals.

**Figure 4:**
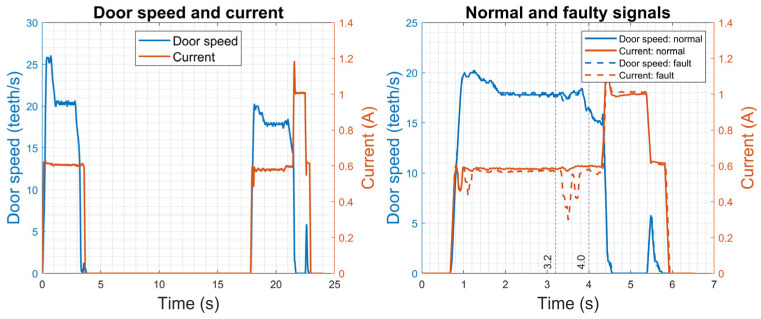
Current signals for door systems, and normal and faulty signals for the closing operation.

Figure 4 presents some examples of signal profiles for both the opening and closing operations. In the opening profile, there is a steady increase in both the speed and current up to a peak, and then a gentle curve and a decline to zero. The closing profile exhibits a pattern similar to the opening profile, but with two notable differences in the current: firstly, the peak current for the closing process is lower than that for the opening process, and secondly, there is a sharp change towards the end of the closing profile. This is accompanied by a minor increase in the speed, which enables the door to reach its fully closed position, where the locking mechanism can be activated [38]. It should be noted that specific types of faults cannot be identified in this dataset [37]. The experimental current signals from the linear actuator in the three fault modes (lack of lubrication, spalling, and backlash) were compared with the faulty signals from railway door systems. However, none of these fault modes showed a similarity to the faulty signals observed in railway door operations. This suggests that the faulty behaviour detected in train doors could be attributed to the multiple fault modes of the numerous components in the train door system.

In this study, current signals from closing operations were employed to detect faults. Examples of normal and faulty signals for the closing operation are shown in Figure 4. In the normal current signal, there are flat curves between 3.2 s and 4.0 s, in contrast to the negative peaks and variations observed in the plot of the faulty data. It is noteworthy that the characteristics of the faults in these door systems are different from those observed in a linear actuator test rig, as explained in Section 2.1.1. Although a specific faulty mode is used as an example, it is noteworthy that the proposed method aims to be universally applicable across various types of fault modes, as it does not rely on assumptions specific to any particular fault mode.

### 2.2. Proposed Methodology

**Figure 5:**
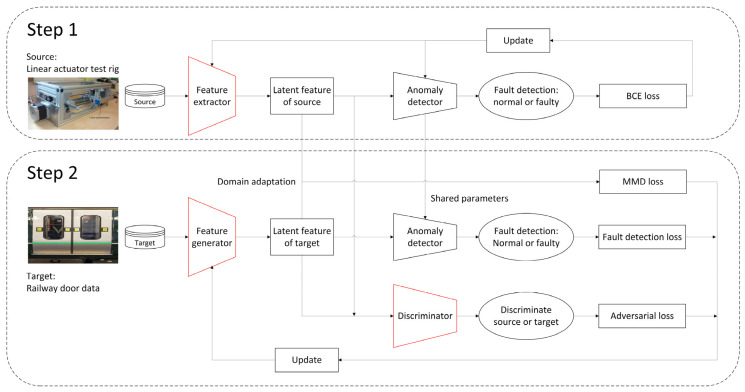
Workflow for the proposed methodology.

The workflow for the proposed methodology for railway door systems, based on one-sided DA with a GAN, is shown in Figure 5. The workflow is divided into two procedures, marked Steps 1 and 2. The source and target domain datasets can be described as follows:

(1) $$D_{s}={\left\{\left(x_{i}^{s},\;y_{i}^{s}\right)\right\}}_{i=1}^{n_{s}}$$

(2) $$D_{t}={\left\{\left(x_{i}^{t},\;y_{i}^{t}\right)\right\}}_{i=1}^{n_{t}}$$

where *D_s_* and *D_t_* are the source and target domain datasets, ***x****^s^* and ***x****^t^* are the source and target feature vectors, *y^s^* and *y^t^* are the source and target labels (which are used to categorise the data into two states: normal or faulty), and *n_s_* and *n_t_* are the numbers of the source and target samples, respectively.

#### 2.2.1. Step 1: Train a Feature Extractor and an Anomaly Detector on the Source Dataset

A feature extractor and an anomaly detector, with the network architectures and hyperparameters given in Table 1 and Table 2, were trained using only the source domain dataset. In order to train the models, a binary cross-entropy (BCE) loss was used as a loss function for both the feature extractor and the anomaly detector. The BCE loss and the optimisation objective are expressed as follows:

(3) $$L_{a}=-\frac{1}{n_{s}}\sum_{i=1}^{n_{s}}\left[y_{i}^{s}ln\left\{f_{a}\left(f_{e}\left(x_{i}^{s}\right)\right)\right\}+\left(1-y_{i}^{s}\right)ln\left\{1-f_{a}\left(f_{e}\left(x_{i}^{s}\right)\right)\right\}\right]$$

(4) $$\left(\theta_{e}^{*},\theta_{a}^{*}\right)={argmin}_{\theta_{e}^{*},\theta_{a}^{*}}\left(L_{a}\right)$$

where *L_a_* is a loss function for both the feature extractor and anomaly detector; *f_e_* and *f_a_* are functions of the feature extractor and the anomaly detector, respectively, which are parameterised by *θ_e_* and *θ_a_*; and θ^*^ is the optimised value of *θ*. Notably, *f_e_* is a mapping function, whereas *f_a_* is a classifier. As shown in Equation (4), the optimisation objective for both the feature extractor and the anomaly detector is to minimise *L_a_*. In view of this optimisation objective, the feature extractor is forced to generate features that are separable by the anomaly detector. It is therefore assumed that faulty samples can be detected by the anomaly detector with high accuracy, meaning that the extracted features of the normal and faulty source samples should be distinct in the latent space. Hence, these features are useful in classifying samples into normal or faulty classes.

#### 2.2.2. Step 2: One-Sided DA from the Target Domain to the Source Domain

Once Step 1 is complete, a feature generator and a discriminator are trained in an adversarial manner. The loss function for the feature generator includes three loss items: MMD loss, a fault detection loss, and an adversarial loss, as follows:

(5) $$L_{g}=L_{MMD}+L_{FD}+L_{AD}$$

(6) $$\theta_{g}^{*}={argmin}_{\theta_{g}}\left(L_{g}\right)$$

where *L_MMD_*, *L_FD,_* and *L_AD_* are the MMD loss, the fault detection loss, and the adversarial loss, respectively. *θ_g_* is a learnable parameter of the feature generator *f_g_*.

The MMD is a statistical measure that is used to quantify the dissimilarity between two distributions, and was initially introduced by Gretton et al. [39]. The concept of MMD is closely related to kernel methods, and it has been used in various areas of machine learning, including domain adaptation and generative modelling. The MMD is defined as a squared distance in the reproducing kernel Hilbert space (RKHS), and can be expressed as follows:

(7) $$L_{MMD}={MMD\left(f_{e}\left(x^{s}\right),\;f_{g}\left(x^{t}\right)\right)=\left\|E_{f_{e}\left(x^{s}\right)\sim P}\left[\varphi \left(f_{e}\left(x^{s}\right)\right)\right]-E_{f_{g}\left(x^{t}\right)\sim Q}\left[\varphi \left(f_{g}\left(x^{t}\right)\right)\right]\right\|}_{H}^{2}$$

(8) $$k\left(x,y\right)={\langle \varphi \left(x\right),\varphi \left(y\right)\rangle }_{H}=exp\left(-\frac{{\left\|x-y\right\|}^{2}}{\sigma }\right)$$

(9) $$\begin{array}{c} {\left\|E_{f_{e}\left(x^{s}\right)\sim P}\left[\varphi \left(f_{e}\left(x^{s}\right)\right)\right]-E_{f_{g}\left(x^{t}\right)\sim Q}\left[\varphi \left(f_{g}\left(x^{t}\right)\right)\right]\right\|}_{H}^{2}\\ =E_{f_{e}\left(x^{s}\right),f_{e}\left(x^{s\prime }\right)\sim P}k\left(f_{e}\left(x^{s}\right),f_{e}\left(x^{s\prime }\right)\right)+E_{f_{g}\left(x^{t}\right),f_{g}\left(x^{t\prime }\right)\sim Q}k\left(f_{g}\left(x^{t}\right),f_{g}\left(x^{t\prime }\right)\right)-2E_{f_{e}\left(x^{s}\right)\sim P,f_{g}\left(x^{t}\right)\sim Q,}k\left(f_{e}\left(x^{s}\right),f_{g}\left(x^{t}\right)\right) \end{array}$$

where *f_e_*(***x****^s^*) and *f_g_*(***x****^t^*) are the latent features from the distributions *P* and *Q*, H represents RKHS using kernel *k*, and *φ* is a mapping function to RKHS. A radial basis function (RBF) kernel, also known as a Gaussian kernel, is chosen as *k* in this research, and σ is set to one. The RBF kernel enables computing the kernel function *k* directly without explicitly knowing the form of *φ*, which is known as the kernel trick.

It is noteworthy that the MMD loss measures the discrepancy between the two latent feature distributions of the source and target domain, which are *f_e_*(***x****^s^*) and *f_g_*(***x****^t^*). The latent features of the source domain are extracted by the feature extractor, which is built in Step 1 and fixed during the training process in Step 2. This means that the source latent features remain invariant in Step 2. In contrast, the feature generator is trained to generate target latent features that are as identical as possible to the source latent features, in order to minimise the MMD loss.

The fault detection loss in Step 2, on the other hand, is calculated using the BCE loss in Equation (10), using the anomaly detector with only target data, as shown in the following equation:

(10) $$L_{FD}=-\frac{1}{n_{t}}\sum_{i=1}^{n_{t}}\left[y_{i}^{t}ln\left\{f_{a}\left(f_{g}\left(x_{i}^{t}\right)\right)\right\}+\left(1-y_{i}^{t}\right)ln\left\{1-f_{a}\left(f_{g}\left(x_{i}^{t}\right)\right)\right\}\right]$$

The anomaly detector *f_a_* built in Step 1 is employed and fixed while training the feature generator, meaning that only the feature generator is trained to minimise *L_FD_*. Hence, once training has been conducted, the anomaly detector should also accurately classify normal and faulty target samples.

The discriminator model is a classifier, and aims to distinguish whether the input sample is a source or target sample. It is trained using both the latent source and target features. A unified dataset *D*′ is defined, which encompasses both the source and target latent features, as follows:

(11) $$D^{\prime }={\left\{\left(z_{j},\;l_{j}\right)\right\}}_{j=1}^{n_{s}+n_{t}}$$

(12) $$\left(z_{j},\;l_{j}\right)=\left\{\begin{array}{c} \left(f_{e}\left(x_{j}^{s}\right),\;l_{s}\right)\;if\;j\;\leq \;n_{s},\;where\;l_{s}\;denotes\;the\;label\;‘source’\\ \left(f_{g}\left(x_{j}^{t}\right),\;l_{t}\right)\;if\;j>n_{s},\;where\;l_{t}\;denotes\;the\;label\;‘target’ \end{array}\right.$$

The discriminator loss for the discriminator and the adversarial loss for the generator are expressed as in the following equations:

(13) $$L_{D}=-\frac{1}{n_{s}+n_{t}}\sum_{j=1}^{n_{s}+n_{t}}\left[l_{j}ln\left\{f_{d}\left(z_{j}\right)\right\}+\left(1-l_{j}\right)ln\left\{1-f_{d}\left(z_{j}\right)\right\}\right]$$

(14) $$L_{AD}=-\frac{1}{n_{s}+n_{t}}\sum_{j=1}^{n_{s}+n_{t}}\left[l_{j}ln\left\{1-f_{d}\left(z_{j}\right)\right\}+\left(1-l_{j}\right)ln\left\{f_{d}\left(z_{j}\right)\right\}\right]$$

(15) $$\theta_{d}^{*}={argmin}_{\theta_{d}}\left(L_{D}\right)$$

where *L_D_* is the discriminator loss, and *f_d_* is a discriminator function parameterised by *θ_d_*. The objective of the discriminator is to minimise the BCE loss for the discriminator, as described in Equation (15). In contrast, Equations (6) and (14) show that the feature generator is trained to maximise the discriminator loss, as *L_D_* should be a maximum when *L_AD_* is minimised. Thus, the optimisation goal for the feature generator is to fool the discriminator, whereas the discriminator is trained to distinguish between the source and target samples in an adversarial manner. The purpose of employing the adversarial loss is to ensure that the latent source and target features are identical to each other.

To summarise, the purposes of each loss item described in Equation (5) are as follows:

- *L_MMD_* forces the feature generator to create target latent features that are as identical as possible to the source latent features as a DA capability.
- *L_FD_* enables normal and faulty target samples to be distinctive and to be detected by the same anomaly detector trained on a rich source domain dataset.
- *L_AD_* ensures that the latent source and target features are identical.

The model built using the proposed method was a simple neural network-based model, as shown in Table 1 and Table 2; however, any other DL model architecture, such as CNN and LSTM, could be employed as long as they have equivalent loss functions. The optimisation of each type of DL model architecture falls outside the scope of this paper.

As a DA method, the proposed methodology offers tremendous advantages from the perspective of fault detection as follows:

1. The latent features of the normal and faulty source samples can be distinguished because the anomaly detector is used to train the feature extractor using normal and faulty source data, which is beneficial for a subsequent one-sided DA.
2. The target domain distribution is aligned toward the source domain distribution on the latent space in a one-sided way, using the anomaly detector built in Step 1.
3. The one-sided DA using the anomaly detector enables normal and faulty target samples to be distinctive, and to be detected by the same anomaly detector trained on a rich source domain dataset.
4. Our approach ensures that the latent source and target features are identical to each other by employing the adversarial training process and a few faulty samples.

The identification of three key research gaps is detailed in Section 1. The resolution of the first and second gaps is achieved through the first, second, and third advantages of our proposed method. The fourth advantage specifically addresses the challenges presented by the third research gap. A quantitative validation of how these advantages effectively address the respective gaps is provided in Section 3. Therefore, the advantages described above enable fault detection across different domains with high reliability and bridge the gap between theoretical domain adaptation research and tangible industrial applications.

### 2.3. Training and Test Datasets

The training and test datasets are summarised in Table 3. The training dataset was used to build the feature extractor, feature generator, and anomaly detector, as described in Section 2.2; the test dataset was then employed to validate the proposed DA method. The training and test samples were selected randomly from the dataset. It is notable that only 10 normal and 5 faulty target samples were used to train the models.

### 2.4. Validation Performance Metrics

A confusion matrix can be used to evaluate the effectiveness of a fault detection system. This is a two-dimensional table containing the frequencies at which samples in each category are accurately identified or incorrectly labelled as belonging to another category. For binary classification in fault detection, the confusion matrix represents four scenarios: positive (faulty) cases can be either correctly identified or missed, and negative (normal) cases may be accurately identified or missed. These outcomes are characterised as true positive (TP), false negative (FN), true negative (TN), and false positive (FP) rates. These rates make up the confusion matrix, which is then used to calculate three performance indicators that are widely used in the industrial sector [40], as given in the following equations:

(16) $$Precision\;\left(P\right)=\frac{TP}{TP+FP}$$

(17) $$Recall\;\left(R\right)=\frac{TP}{TP+FN}$$

(18) $$F1\;score=\frac{2PR}{P+R}$$

In general, the precision quantifies the proportion of samples correctly predicted as positive, while the recall represents the extent to which positive predictions correctly capture positive samples. Optimising precision and recall involves a trade-off [41]; for example, perfect recall can be achieved by predicting all samples as positive, but this results in very low precision due to numerous false alarms. In contrast, the precision will be perfect if a model predicts only the most likely positive sample as positive and the rest as negative, but this approach will result in a very low recall. One method of considering both precision and recall simultaneously is to compute their harmonic mean, referred to as the F1 score, as given in Equation (18). In this research, the F1 score, which varies from zero to one, is used to assess the fault detection accuracy. A higher F1 score indicates greater accuracy in detecting faults; the opposite is true for a lower score.

### 2.5. Alternative DA Models for Comparison Purposes

In this study, other types of DA models were built for comparison purposes. The following approaches were implemented:

(1) Transfer component analysis (TCA): The primary goal of this approach is to search the feature subspace of different domains (or the source and target domains), where the domain shift between them is minimised [42]. The TCA algorithm tries to learn some transfer components across domains in an RKHS. Within the subspace defined by these transfer components, the characteristics of the data are preserved, and the data distributions across various domains are closely aligned. Logistic regression is selected as the classification method.
(2) DANN: The aim of this approach is to find a new representation of the input features in which the source and target data cannot be distinguished by any discriminator network [28]. This new representation is learned by an encoder network in an adversarial fashion. A task network is trained on the encoded space in parallel to the encoder and discriminator networks.

These models are well-known DA methods, and have been used in many previous research papers as benchmark models. In order to build these two models, a publicly available library called the Awesome Domain Adaptation Python Toolbox (ADAPT) [43] was used. A labelled source dataset and an unlabelled target dataset were used for training, as these two models are based on an unsupervised DA method. In contrast, labelled source and target datasets, which included a few faulty samples, were used to build the proposed model, as shown in Table 3. It could be argued that comparing the proposed model with unsupervised DA methods is unfair; however, to the best of our knowledge, there is no representative DA model similar to ours. In order to make the comparative study fairer, the number of available target samples were increased, as shown in Table 4, compared to the dataset for the proposed method, shown in Table 3. The cross-validation, which typically relies on labelled data for evaluating model performance, is not applicable as target training data is unlabelled, as given in Table 4. Therefore, the randomly selected training and test samples were used, as is explained in Section 2.3.

**Figure 6:**
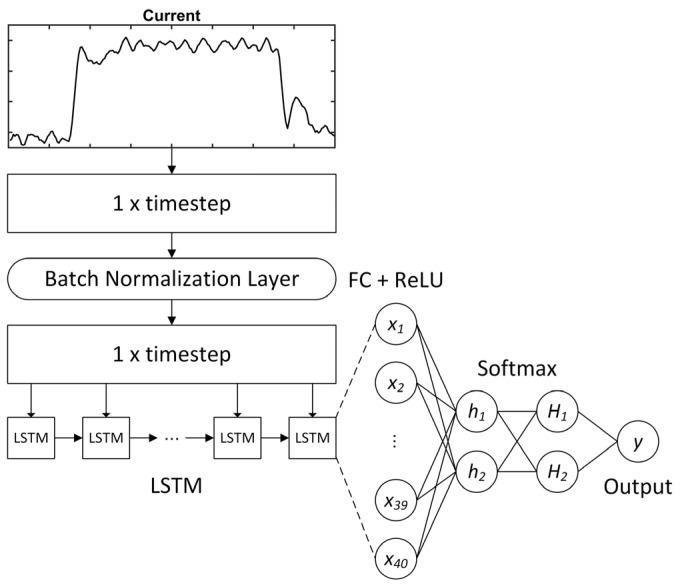
DL model from scratch [44].

A third model was also built, as follows:

(3) DL model from scratch: This model was trained using only the target training dataset summarised in Table 3, in which there were 10 normal and five faulty samples. Figure 6 and Table 5 show the network architecture and hyperparameters for the model, which was previously used as a comparative model in [44].

### 2.6. Sensitivity Analysis

Sensitivity analysis is a common practice in technological fields, and is carried out to examine how variations in model parameters affect the output of a model [45]. To explore the sensitivity of the model to the probability threshold, a receiver operating characteristic (ROC) curve is employed. A ROC gives a comprehensive overview of the trade-off between the false positive rate (FPR) and true positive rate (TPR). The optimal ROC curve has an FPR of zero and a TPR of one. A metric known as the area under the ROC Curve (AUC) is determined by calculating the area under the complete ROC curve between (0, 0) and (1, 1). The AUC offers a single-value overview of the ROC curve, and has a value of one in the case of an ideal ROC curve. In this research, the ROC curve was plotted to correspond to the probability threshold to detect faulty samples. The probability was determined using the sigmoid function of the anomaly detector, as given in Table 1.

## 3. Results and Discussion

### 3.1. Performance Metrics and Sensitivity Analysis

**Figure 7:**
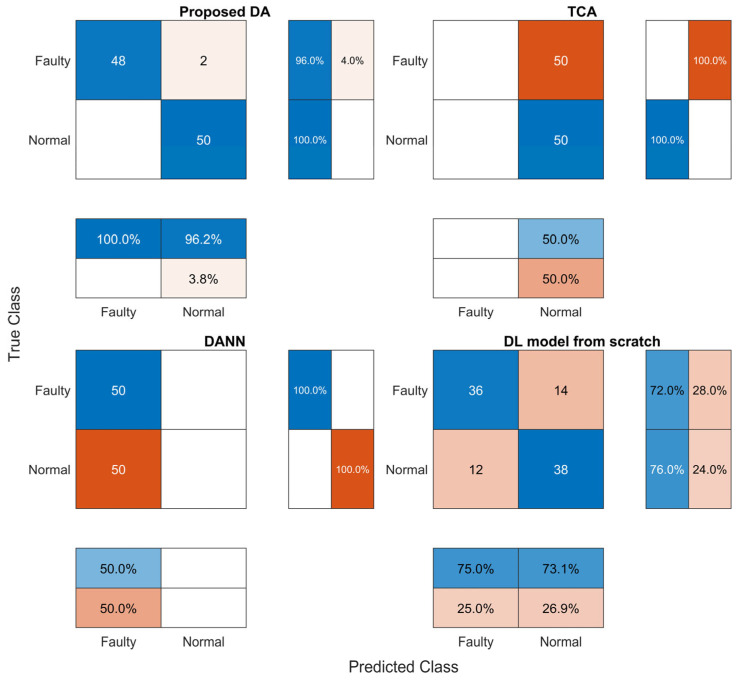
Confusion matrix.

The performance metrics and confusion matrices are given in Table 6 and Figure 7. The highest fault detection accuracy was achieved by the proposed method, with an F1 score of 97.9%, while DANN and the DL model from scratch had considerably lower fault detection accuracies, with F1 scores of 66.6% and 73.4%, respectively. The precision and F1 score for TCA could not be calculated, as all of the predictions were normal, as shown in Figure 7. The results reveal that a fault detection model for the target domain, which in this paper was a railway door system, can be built accurately by our DA method using source domain data from a linear actuator test rig dataset.

**Figure 8:**
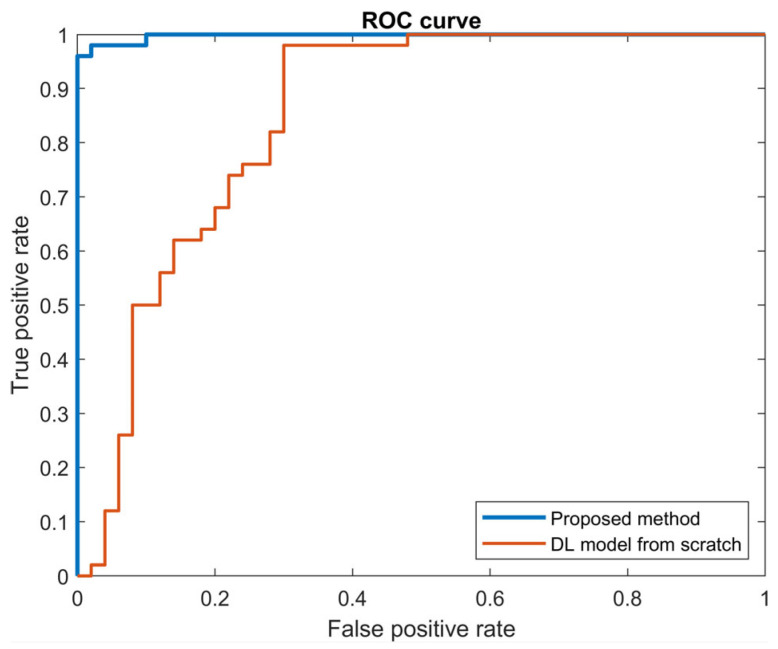
ROC curves.

However, it is also necessary to ensure that the fault detection model does not have a high sensitivity to the probability threshold that is used to determine whether or not a sample is faulty. A sensitivity analysis was performed only for the proposed DA method and the DL model from scratch, as there was no need for a sensitivity analysis of TCA and DANN in view of the low fault detection accuracy (Table 6). As shown in Figure 8 and Table 7, the ROC curve for the proposed DA model was much closer to the ideal ROC curve than that of the DL model from scratch. The values of AUC for the proposed DA and DL models from scratch were 0.9976 and 0.8484, respectively. The ROC and AUC results indicate that the proposed DA method was the most accurate and the least sensitive to the threshold, and hence the most robust against variation in the input features. The proposed DA model was therefore the most accurate and robust of all the alternative models.

### 3.2. t-SNE Visualisation

**Figure 9:**
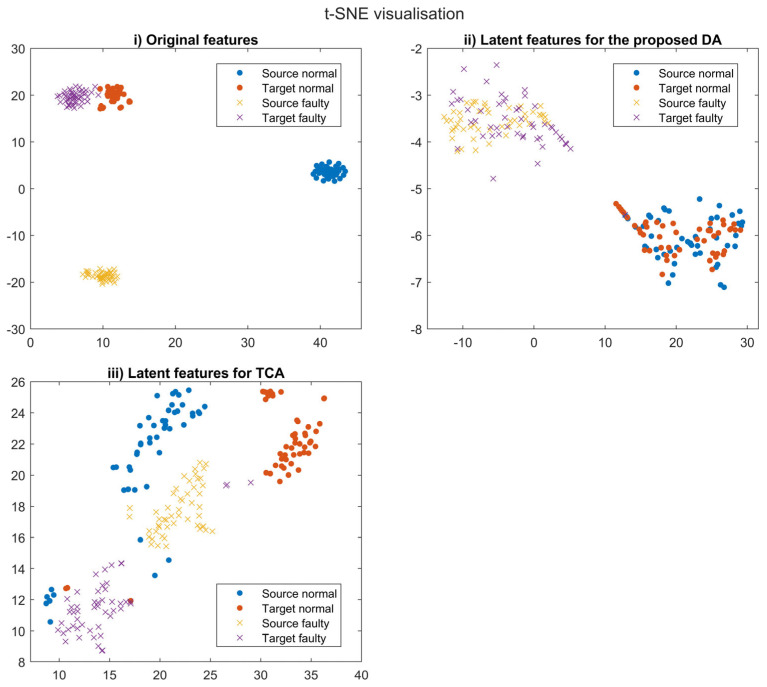
t-SNE visualisation: (**i**) original features; (**ii**) latent features for the proposed method; (**iii**) latent features for TCA.

The feature distributions of the test dataset were visualised with t-SNE, as shown in Figure 9. Four distinct distributions (relating to the source normal, source faulty, target normal, and target faulty samples) can be found in the original feature space, as illustrated in Figure 9, due to the domain shift. Suppose a fault detection model is built based on traditional ML and DL by using source domain data. In that case, fault detection accuracy should be considerably degraded when the model is applied to target domain data due to domain shift. The assumption underlying traditional ML and DL approaches is that the distribution of the test data is identical to that of the training data; however, these distributions may differ due to the different components and detailed specifications of the machinery, as clearly seen in Figure 9. In addition, traditional ML and DL typically do not possess DA capabilities. In contrast, the latent features of the normal and faulty target distributions were aligned toward the normal and faulty source distributions in the proposed method, as can be seen from Figure 9. A significant finding was that the aligned normal and faulty samples were sufficiently distinctive to be classified by the fault detection model. This clear distinction between the normal and faulty samples can be attributed to the anomaly detector that was employed to separate the two distributions, while the feature generator was trained to align the source and target data. Thus, the anomaly detector can detect faulty target samples with a high level of accuracy, as shown by the qualitative performance validation in Table 6.

However, although each feature distribution of TCA could be closer than the original features, these were unaligned, as shown in Figure 9. The poor fault detection accuracy of TCA, as shown in Table 6, may be due to this misalignment between the two domains. The misalignment of TCA and DANN may also be related to the level of similarity between the source and target domain, which might be insufficient for these models. The low similarity can be attributed to the different components involved, and specifically to railway door systems and the linear actuator test rig. These methods are therefore incapable of correctly adapting the source and target domains to become identical, despite being successful candidates as representative DA methods. Thus, the proposed DA method enables alignment between two distributions as well as clear separation between normal and faulty samples, even though the similarity is relatively low, whereas other models are unable to align the two.

### 3.3. Limitations

It should be emphasised that a small number of target faulty samples need to be employed with the proposed method, which means that our methodology is not unsupervised DA. In addition, only one application was used to validate the performance of the model, and further validation may be needed in the future. However, this research shows that the two domains can be aligned even when the level of similarity is low and only a few faulty samples in the target domain are used. Thus, our method is reliable and applicable to real-world industrial settings.

## 4. Conclusions

A novel fault detection approach based on one-sided DA using GANs for railway door systems has been proposed. In this study, the source and target domain data were drawn from a linear actuator test rig and a real-world railway door, respectively. Firstly, the anomaly detector and feature generator were trained using the label-rich source domain data to generate distinctive source latent features, and the target domain data were then aligned with the latent source features in a one-sided way. To the best of our knowledge, this is the first paper to propose a fault detection approach based on DA for railway door systems.

As a result, the performance metrics and sensitivity analysis results showed that the proposed method is the most accurate, with an F1 score of 97.9%, and is also the most robust against variation in the input features. Thus, the proposed method enables faulty target samples to be aligned with the source samples and detected accurately by the same anomaly detector, which is built with rich source data. This results in high reliability of fault detection for real-world applications despite the low level of similarity between different domains. Hence, the proposed method is not only the most accurate and robust compared to alternative models but also bridges the gap between theoretical domain adaptation research and tangible industrial applications.

In future research, it would be valuable to quantify the similarity between domains in order to be able to apply DA methods while maintaining high reliability. This is because the degree of similarity between the two domains that is required in order to be able to apply the DA method successfully is unclear. The method proposed in this research used a few faulty samples from a target domain to tackle this issue; however, even a few faulty samples from a target domain may sometimes be unavailable. An unsupervised DA method would then need to be employed, in which case the required degree of similarity between the two domains would be unknown. Addressing these issues could represent a direction for future work.

## Figures and Tables

**Table 1:** Network architecture and numbers of learnable parameters.

Model	Layer	Output Shape	Parameters
Feature extractor	Linear	1 × 100	20,000
	ReLU	1 × 100	-
	Linear	1 × 50	5050
	ReLU	1 × 50	0.0001
	Linear	1 × 10	510
Anomaly detector	Linear	1 × 5	55
	ReLU	1 × 5	-
	Linear	1 × 1	6
	Sigmoid	1 × 1	-
Feature generator	Linear	1 × 100	20,000
	ReLU	1 × 100	-
	Linear	1 × 50	5050
	ReLU	1 × 50	0.0001
	Linear	1 × 10	510
Discriminator	Linear	1 × 3	33
	ReLU	1 × 3	-
	Linear	1 × 1	4
	Sigmoid	1 × 1	-

**Table 2:** Hyperparameters for the models.

Model	Hyperparameter Name	Hyperparameter
Feature extractor	Optimiser	Adam
	Learning rate	0.001
	Max epoch	5000
Anomaly detector	Optimiser	Adam
	Learning rate	0.001
	Max epoch	5000
Feature generator	Optimiser	Adam
	Learning rate	0.001
	Max epoch	8000
Feature generator	Optimiser	Adam
	Learning rate	0.1
	Max epoch	8000

**Table 3:** Training and test datasets.

Training/Test	Domain	Normal	Faulty	Total
Training	Source	50	50	100
Training	Target	10	5	15
Test	Target	50	50	100

**Table 4:** Training and test datasets for comparison models.

Training/Test	Domain	Label	Normal	Faulty	Total
Training	Source	Labelled	50	50	100
Training	Target	Unlabelled	100		100
Test	Target	Labelled	50	50	100

**Table 5:** Hyperparameters of a fault detection model for the DL model from scratch [44].

Layer	Hyperparameter Name	Hyperparameter
Whole layers	Optimiser	Adam
	Max epoch	3000
	Mini-batch size	120
	Learning rate	0.0001
LSTM	Activation function for the hidden state	Tanh
	Activation function for the gates	Sigmoid
	Number of activation units	40
FC	Number of hidden units	2

**Table 6:** Fault detection accuracy.

Model	Precision (%)	Recall (%)	F1 Score (%)
Proposed DA model	100	96	97.9
TCA	NaN	50	NaN
DANN	50	100	66.6
DL model from scratch	75.0	72.0	73.4

**Table 7:** AUC values.

Model	Proposed Method	DL Model from Scratch
AUC	0.9976	0.8484

## Data Availability

Publicly available datasets were analyzed in this study. This data can be found here: https://figshare.com/s/dac98f9c1bc46b7a2800 (accessed on 2 November 2023).
